# Protective Efficacy of Lyophilized Vesicular Stomatitis Virus–Based Vaccines in Animal Model

**DOI:** 10.3201/eid3005.231248

**Published:** 2024-05

**Authors:** Abd’jeleel Salawudeen, Geoff Soule, Nikesh Tailor, Levi Klassen, Jonathan Audet, Angela Sloan, Yvon Deschambault, David Safronetz

**Affiliations:** University of Manitoba, Winnipeg, Manitoba, Canada (A. Salawudeen, D. Safronetz);; Public Health Agency of Canada, Winnipeg (G. Soule, N. Tailor, L. Klassen, J. Audet, A. Sloan, Y. Deschambault, D. Safronetz)

**Keywords:** vesicular stomatitis virus, vaccines, Ebola virus, Lassa virus, glycoproteins, lyophilized, guinea pigs, animal models, viruses, zoonoses

## Abstract

We evaluated the in vitro effects of lyophilization for 2 vesicular stomatitis virus–based vaccines by using 3 stabilizing formulations and demonstrated protective immunity of lyophilized/reconstituted vaccine in guinea pigs. Lyophilization increased stability of the vaccines, but specific vesicular stomatitis virus–based vaccines will each require extensive analysis to optimize stabilizing formulations.

Live recombinant vesicular stomatitis virus (VSV) expressing the Ebola virus (EBOV) glycoprotein (VSV∆G/EBOVGP) was evaluated during 2014–2015 as a vaccine to limit the effects of EBOV disease (*1*). Because of the success and safety of the EBOV vaccine, similar VSV-based vaccines have been proposed for Sudan and Marburg viruses and for other etiologic agents of viral hemorrhagic fever diseases, such as Lassa virus (LASV) (*2*).

Cold chain maintenance for distributing and storing VSV-based vaccines is a logistical challenge, especially when ultralow temperatures (−60°C to −80°C) are required. The challenge is greater in rural areas, particularly in developing countries, where infrastructure and transport systems are often deficient. We evaluated the effects of lyophilization on the in vitro recoverability and in vivo protective efficacy of VSV-based vaccines.

We conducted animal studies in accordance with the Canadian Council of Animal Care guidelines; studies received approval from the Canadian Science Centre for Human and Animal Health’s institutional Animal Care and Use Committee. We performed work involving infectious Lassa virus in a Biosafety Level 4 laboratory within the Public Health Agency of Canada. When required, we inactivated materials for subsequent analysis according to approved procedures.

## The Study

We conducted propagation and titration (50% tissue culture infectious dose [TCID_50_]) of VSV∆G/EBOVGP and VSV-based LASV (VSV∆G/LASVGPC) vaccines by using Vero E6 cells as previously described (*3*). We evaluated 4 excipients as stabilizers: 2.5% lactalbumin hydrolysate (L), 5% sucrose (S), 2.5% trehalose (T), and 0.25% gelatin (G). We prepared 2× concentrations of each solution initially in Hanks’ balanced salt solution and then evaluated 3 combinations (LS, LST, or LSTG) (*4*,*5*). The control formulation for lyophilization was Dulbecco modified Eagle medium (DMEM) without additives. We mixed each excipient combination 1:1 with VSV∆G/EBOVGP (stock titer 1.26 × 10^7^ TCID_50_/mL) or VSV∆G/LASVGPC (2.83 × 10^7^ TCID_50_/mL) and dispensed 200 µL of the mixture into 4 mL sterile glass vials (Electron Microscopy Sciences, https://www.emsdiasum.com). We lyophilized the vaccine mixtures by using an automated FreeZone Triad Benchtop Freeze Dryer (Labconco, https://www.labconco.com) according to the manufacturer’s specifications (Appendix Table).

**Figure 1:**
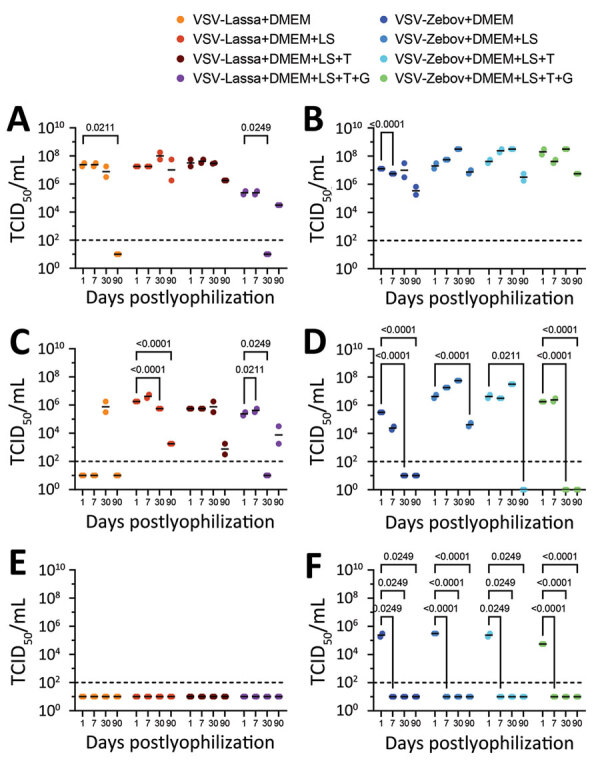
Vaccine recovery after lyophilization in study of protective efficacy of lyophilized vesicular stomatitis virus–based vaccines in animal model. A) VSV∆G/LASVGPC vaccine stored at 4°C; B) VSV∆G/EBOVGP vaccine stored at 4°C; C) VSV∆G/LASVGPC vaccine stored at 21°C; D) VSV∆G/EBOVGP vaccine stored at 21°C; E) VSV∆G/LASVGPC vaccine stored at 21°C; F) VSV∆G/EBOVGP vaccine stored at 21°C. VSV∆G/LASVGPC or VSV∆G/EBOVGP vaccines were lyophilized in DMEM containing no excipients or containing combinations of 5% lactalbumin hydrolysate, 10% sucrose, 5% trehalose, or 0.5% gelatin and stored at different temperatures. At the specified time points, vaccines were resuspended in triplicate in normal saline, titered by using standard tissue culture techniques, and the median TCID_50_ was calculated for each. p values are indicated above brackets. Errors bars are SDs. DMEM, Dulbecco modified Eagle medium; G, gelatin; LS, lactalbumin hydrolysate and sucrose; NC, not calculated; T, trehalose; TCID_50_, 50% tissue culture infectious dose; VSV-Lassa, vesicular stomatitis virus expressing Lassa virus glycoprotein; VSV-Zebov, vesicular stomatitis virus expressing Ebola virus glycoprotein.

We stored the vials at 4°C, 21°C, or 37°C for 1, 7, 30, and 90 days after lyophilization. At those time points, we reconstituted each vaccine/stabilizer combination in triplicate in 200 µL of 0.85% saline for 1 hour at room temperature by using gentle agitation. We then prepared 10-fold serial dilutions in DMEM and determined virus titers by using standard TCID_50_ methodologies, as previously described (*3*). Titrations of formulations conducted immediately before lyophilization indicated that the addition of stabilizers had no adverse effect on vaccine recovery. We performed mean difference calculations to compare TCID_50_ data collected on day 1 and day 90 after lyophilization by using 2-way analysis of variance in GraphPad Prism 10 (Graphpad, https://www.graphpad.com). For the VSV∆G/LASVGPC vaccine, the 3 stabilizer formulations provided consistent levels of virus recovery; we observed little variation after lyophilization/reconstitution and only minor decreases in titers when stored at 4°C (Table 1; Figure 1). The VSV∆G/LASVGPC construct was stable for >90 days. By comparison, the vaccine was not recoverable after >30 days when stored at 4°C without stabilizers (DMEM only). We observed similar patterns of stability for the VSV∆G/LASVGPC vaccine when storage temperatures were increased; albeit, even with the addition of stabilizers, vaccine recovery was immediately impaired by >1 log_10_ when stored at room temperature (21°C), and no recoverable vaccine was observed when formulations were stored at 37°C. The recovery trends for stabilizer formulations and storage temperature were similar for VSV∆G/EBOVGP and VSV∆G/LASVGPC. However, in general, the VSV∆G/EBOVGP vaccine was more stable than the VSV∆G/LASVGPC vaccine even without stabilizing agents or when stored at increased temperatures (Table 1).

**Table 1:** Infectious titers of lyophilized vaccines after 90 day storage at different temperatures in study of protective efficacy of lyophilized vesicular stomatitis virus–based vaccines in animal model*

Vaccine	Lyophilization medium			
	DMEM	DMEM + LS	DMEM + LS +T	DMEM + LS + T + G
VSV∆G/LASVGPC				
4°C	6.36 (5.49–7.23)	0.25 (–0.62 to 1.12)	1.25 (0.38–2.12)	0.88 (0.005–1.75)
21°C	NC	3.00 (2.06–3.94)	2.88 (1.94–3.82)	1.50 (0.56–2.44)
37°C	NC	NC	NC	NC
VSV∆G/EBOVGP				
4°C	1.57 (0.83–2.31)	0.43 (–0.32 to 1.17)	1.13 (0.39–1.87)	1.55 (0.81–2.29)
21°C	4.50 (4.22–4.78)	2.00 (1.72–2.28)	6.63 (6.35–6.91)	6.25 (5.97–6.53)
37°C	4.38 (4.20–4.56)	4.50 (4.32–4.68)	4.38 (4.20–4.56)	3.75 (3.57–3.93)

*Values are no. (95% CI), representing the log_10_ decreases in infectious titers (median 50% tissue culture infectious dose) for vaccines that were lyophilized in the presence of various stabilizers, stored at the indicated temperatures for 90 days, and then reconstituted. Comparisons are between 1 and 90 days after lyophilization. DMEM, Dulbecco modified Eagle medium; G, gelatin; LS, lactalbumin hydrolysate and sucrose; NC, not calculated; T, trehalose; VSV∆G/EBOVGP, vesicular stomatitis virus expressing Ebola virus glycoprotein; VSV∆G/LASVGPC, vesicular stomatitis virus expressing Lassa virus glycoprotein.

**Figure 2:**
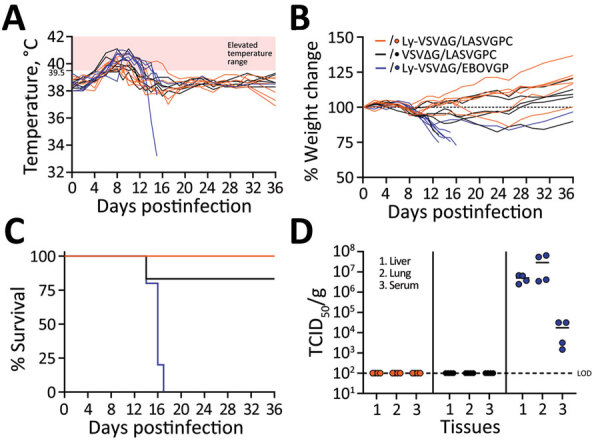
Protective efficacy of lyophilized vesicular stomatitis virus–based vaccines in guinea pig model. A) Body temperatures; B) weight changes; C) survival; D) virus titrations in different tissues. Groups of 10 Hartley guinea pigs each were immunized with VSV∆G/LASVGPC vaccine or lyophilized/reconstituted Ly-VSV∆G/LASVGPC or Ly-VSV∆G/EBOVGP. Ly-VSV∆G/EBOVGP was used as the sham-vaccinated inoculum control group. Animals were challenged 28 days after immunization with a lethal dose of guinea pig–adapted Lassa virus Josiah strain. Disease progression was monitored in 6 animals in each group; the remaining 4 animals per group were euthanized on day 13 postinfection for analysis of infectious Lassa virus in tissues. LOD, limit of detection; Ly-VSV∆G/EBOVGP, lyophilized vesicular stomatitis virus expressing Ebola virus glycoprotein; Ly-VSV∆G/LASVGPC, lyophilized vesicular stomatitis virus expressing Lassa virus glycoprotein; TCID_50_, 50% tissue culture infectious dose; VSV∆G/LASVGPC, vesicular stomatitis virus expressing Lassa virus glycoprotein.

The protective efficacy of VSV∆G/EBOVGP and VSV∆G/LASVGPC vaccines against lethal homologous virus challenge is well established (*6*). To further evaluate lyophilized VSV formulations, we immunized groups of 10 Hartley guinea pigs 1 time with 1 × 10^6^ PFU of either VSV∆G/LASVGPC or lyophilized/reconstituted VSV∆G/LASVGPC (Ly-VSV∆G/LASVGPC) or lyophilized/reconstituted VSV∆G/EBOVGP (Ly-VSV∆G/EBOVGP) via intraperitoneal injection as previously described (*7*). According to in vitro assessments, the lyophilized vaccines contained the LST stabilizer formulation and were stored after lyophilization for 1 week at 4°C. We collected a blood sample from each of the 30 animals at 28 days postimmunization, after which we challenged them with a previously determined lethal dose (10^4^ TCID_50_) or 10× the 50% lethal dose of guinea pig–adapted LASV Josiah strain via intraperitoneal inoculation (*8*). We monitored 6 animals per group for disease progression and survival; we euthanized the remaining 4 animals per group on postinfection day 13 to analyze virus titers in tissue samples. The first signs of infection developed on postinfection day 8; increased body temperatures near 40°C occurred in most animals (Figure 2, panel A). Body temperatures in animals immunized with VSV∆G/LASVGPC or Ly-VSV∆G/LASVGPC returned to normal within 2–3 days, whereas body temperatures in animals that received Ly-VSV∆G/EBOVGP remained elevated at 40°C–41°C until death of those animals, which occurred 14–16 days postinfection. We observed weight loss >12% only in Ly-VSV∆G/EBOVGP immunized animals (control group); consistent weight losses occurred during 8–10 days postinfection (Figure 2, panel B). One animal immunized with VSV∆G/LASVGPC experienced an abrupt drop in body weight requiring humane euthanasia on day 13 postinfection. Overall, 100% (6/6) of animals immunized with Ly-VSV∆G/LASVGPC and 83.3% (5/6) immunized with VSV∆G/LASVGPC survived the LASV challenge compared with 16.6% (1/6) in the Ly-VSV∆G/EBOVGP control group (Figure 2, panel C). Supporting the survival data, we only found infectious LASV in tissues collected on postinfection day 13 from the Ly-VSV∆G/EBOVGP–immunized control animals (Figure 2, panel D). Although not tested in vivo, the in vitro data supports similar protective responses from lyophilized VSV∆G/LASVGPC stabilized with LST or LS formulations for >30 days at 21°C or 90 days at 4°C.

We evaluated vaccine-induced humoral immune responses in serum samples collected immediately before virus challenge (28 days postimmunization) by using LASV and EBOV glycoprotein-specific ELISAs, as previously described (*8*,*9*). Animals immunized with Ly-VSV∆G/EBOVGP vaccine all had EBOV-specific ELISA titers >1:6,400. Although those animals were not challenged with EBOV to assess the in vivo protective efficacy of the Ly-VSV∆G/EBOVGP vaccine, their antibody responses were consistent with a predicted protective response on the basis of findings from other studies, including studies using a similar EBOV guinea pig model (*9*,*10*). Instead, we used Ly-VSV∆G/EBOVGP–immunized animals as sham-vaccinated control animals in the lethal LASV challenge experiment to control for nonspecific immunity associated with the LST stabilizer formulation. We monitored LASV-specific responses by using a glycoprotein ELISA developed for use in humans (Zalgen Labs, https://www.zalgen.com), which impedes direct determination of antibody concentrations in guinea pig samples. Nevertheless, we observed >75-fold increases in seroreactivity according to optical densities and average calculated concentrations in animals immunized with the Ly-VSV∆G/LASVGPC or VSV∆G/LASVGPC vaccines compared with preimmunization samples or serum samples collected from animals immunized with Ly-VSV∆G/EBOVGP (Table 2). Furthermore, the similar average values calculated for animals immunized with Ly-VSV∆G/LASVGPC and VSV∆G/LASVGPC indicates the lyophilization process did not appear to deleteriously effect the overall immunogenicity of the VSV-LASV vaccine.

**Table 2:** Serologic evaluation of Lassa virus antibodies in immunized guinea pigs in study of protective efficacy of lyophilized vesicular stomatitis virus–based vaccines in an animal model*

Animal group, n = 10 each	Median (range)	Average (SEM)	Fold increase†
Preimmunization	1.4 (0.8–3.8)	1.8 (0.4)	NA
Ly-VSV∆G/LASVGPC	100.7 (8–373)	148.6 (44.2)	82
VSV∆G/LASVGPC	160.5 (79–262.8)	172 (17.7)	95
Ly-VSV∆G/EBOVGP	2 (0.7–6.1)	2.4 (0.6)	1.4

*Serum was analyzed for the presence of Lassa glycoprotein-specific antibodies by using an ELISA before challenge with Lassa virus. The standard curve was generated by using human control serum samples; therefore, values should be considered relative and for comparison purposes only. Ly-VSV∆G/EBOVGP, lyophilized vesicular stomatitis virus expressing Ebola virus glycoprotein; Ly-VSV∆G/LASVGPC, lyophilized vesicular stomatitis virus expressing Lassa virus glycoprotein; NA, not applicable; VSV∆G/LASVGPC, vesicular stomatitis virus expressing Lassa virus glycoprotein. †Fold change of average values from immunized animals compared with randomized preimmunization control specimens.

## Conclusions

We show that lyophilization can increase stability of VSV-based vaccines, potentially enhancing infrastructure and transport systems in rural areas and developing countries where cold chain management is challenging. Although the 2 VSV-based vaccines evaluated in this study only varied in their glycoproteins, in vitro recoverability efficiencies between them using different stabilizers, particularly gelatin, imply that a universal lyophilization method for all VSV-based vaccines might not be achievable. Therefore, each VSV-based vaccine will require in-depth experimentation to optimize formulations.

Appendix Additional information for protective efficacy of lyophilized vesicular stomatitis virus–based vaccines in animal model.

## References

[1] Henao-Restrepo AM, Camacho A, Longini IM, Watson CH, Edmunds WJ, Egger M, et al. Efficacy and effectiveness of an rVSV-vectored vaccine in preventing Ebola virus disease: final results from the Guinea ring vaccination, open-label, cluster-randomised trial (Ebola Ça Suffit!). Lancet. 2017;389:505–18. 10.1016/S0140-6736(16)32621-6 28017403 PMC5364328

[2] Fathi A, Dahlke C, Addo MM. Recombinant vesicular stomatitis virus vector vaccines for WHO blueprint priority pathogens. Hum Vaccin Immunother. 2019;15:2269–85. 10.1080/21645515.2019.1649532 31368826 PMC6816421

[3] Garbutt M, Liebscher R, Wahl-Jensen V, Jones S, Möller P, Wagner R, et al. Properties of replication-competent vesicular stomatitis virus vectors expressing glycoproteins of filoviruses and arenaviruses. J Virol. 2004;78:5458–65. 10.1128/JVI.78.10.5458-5465.2004 15113924 PMC400370

[4] Kang MS, Jang H, Kim MC, Kim MJ, Joh SJ, Kwon JH, et al. Development of a stabilizer for lyophilization of an attenuated duck viral hepatitis vaccine. Poult Sci. 2010;89:1167–70. 10.3382/ps.2009-00620 20460663

[5] Sarkar J, Sreenivasa BP, Singh RP, Dhar P, Bandyopadhyay SK. Comparative efficacy of various chemical stabilizers on the thermostability of a live-attenuated peste des petits ruminants (PPR) vaccine. Vaccine. 2003;21:4728–35. 10.1016/S0264-410X(03)00512-7 14585683

[6] Liu G, Cao W, Salawudeen A, Zhu W, Emeterio K, Safronetz D, et al. Vesicular stomatitis virus: from agricultural pathogen to vaccine vector. Pathogens. 2021;10:1092. 10.3390/pathogens10091092 34578125 PMC8470541

[7] Stein DR, Sroga P, Warner BM, Deschambault Y, Poliquin G, Safronetz D. Evaluating temperature sensitivity of vesicular stomatitis virus-based vaccines. Emerg Infect Dis. 2019;25:1563–6. 10.3201/eid2508.190281 31141474 PMC6649338

[8] Safronetz D, Rosenke K, Westover JB, Martellaro C, Okumura A, Furuta Y, et al. The broad-spectrum antiviral favipiravir protects guinea pigs from lethal Lassa virus infection post-disease onset. Sci Rep. 2015;5:14775. 10.1038/srep14775 26456301 PMC4600983

[9] Cao W, He S, Liu G, Schulz H, Emeterio K, Chan M, et al. The rVSV-EBOV vaccine provides limited cross-protection against Sudan virus in guinea pigs. NPJ Vaccines. 2023;8:91. 10.1038/s41541-023-00685-z 37301890 PMC10257645

[10] Marzi A, Engelmann F, Feldmann F, Haberthur K, Shupert WL, Brining D, et al. Antibodies are necessary for rVSV/ZEBOV-GP-mediated protection against lethal Ebola virus challenge in nonhuman primates. Proc Natl Acad Sci U S A. 2013;110:1893–8. 10.1073/pnas.1209591110 23319647 PMC3562844

