# Supplementary material for: Protective Efficacy of Lyophilized Vesicular Stomatitis Virus–Based Vaccines in Animal Model
Source: Emerg Infect Dis. 2024 May;30(5):1004–8. doi: 10.3201/eid3005.231248 (PMC11060439; doi:10.3201/eid3005.231248)
Supplement: Appendix — Additional information for protective efficacy of lyophilized vesicular stomatitis virus–based vaccines in animal model. [file 23-1248-Techapp-s1.pdf]

Article DOI: https://doi.org/10.3201/eid3005.231248

EID cannot ensure accessibility for supplementary materials supplied by authors. Readers who have difficulty accessing supplementary content should contact the authors for assistance.

Protective Efficacy of Lyophilized Vesicular Stomatitis–Based Virus Vaccines in Animal Model

Appendix

Appendix Table. Lyophilization program*

Cycle	Duration, min	Shelf temperature, °C	Vacuum pressure, mbar
Freezing	230	–40	0.53
Primary drying	60	–35	0.53
	60	–30	0.53
	60	–25	0.53
	60	–20	0.53
	60	–15	0.53
	60	–10	0.53
	60	–5	0.53
	60	0	0.53
	40	5	0.53
	20	10	0.53
	20	15	0.53
	20	20	0.53
	20	25	0.53
	25	30	0.53
Secondary drying	30	30	0.13

*Vaccine mixtures were lyophilized by using an automated FreeZone Triad Benchtop Freeze Dryer (Labconco, https://www.labconco.com).
